# Retraction: A Short Caspase-3 Isoform Inhibits Chemotherapy-Induced Apoptosis by Blocking Apoptosome Assembly

**DOI:** 10.1371/journal.pone.0306366

**Published:** 2024-06-27

**Authors:** The PLOS ONE Editors

Following the publication of this article [1], concerns were raised regarding results presented in Figs 1, 2, 5 and S1. Specifically,

- In the Pro-Caspase-3 panel of Figs 1 and S1, when levels are adjusted to view the backgrounds, the 72 hours lane appears more similar to the 96 hours lane than would be expected from independent results.
- In Fig 2A, the following bands appear similar:
  ○ ProC3-GFP panel, lanes 1, 2, 4, and 8
  ○ ProC3-GFP panel, lanes 3 and 6
  ○ β-actin panel, lanes 2 and 3
  ○ β-actin panel, lanes 6 and 7
- Fig 2C, α-Fodrin panel, lane 1 appears similar to lane 4 when flipped horizontally.
- Fig 2D, α-Fodrin panel, lane 1 appears similar to lane 4 when flipped horizontally.
- In Fig 5C, the following bands appear similar:
  ○ Active caspase-9 panel, lanes 6 and 8
  ○ Procaspase-3, lanes 1 and 4, when levels are adjusted to view the backgrounds
  ○ C3s-YFP panel, lanes 1 and 4
  ○ C3s-YFP panel, lanes 2 and 8
- In Fig 5D, the following bands appear similar:
  ○ Procaspase-3 panel, lanes 2 and 8
  ○ Procaspase-3 panel, lanes 4 and 6 when flipped horizontally
- When levels are adjusted, irregularities were detected in the background of the following panels:
  ○ Fig 2A, (Pro)C3-GFP panel
  ○ Fig 2B, 2C, and 2D (Cleaved) α-Fodrin panels, between lanes 3 and 4
  ○ Fig 5A (Pro)Caspase-3 and C3s-YFP panels
  ○ Fig 5B (C3s-)YFP and Procaspase-3 panels
  ○ Fig 5C Procaspase 3 and C3s-YFP panels
  ○ Fig 5D (Pro)caspase 3 and Active Caspase 9 panels
  ○ Fig 5E (C3s-)YFP, Active caspase 9, and Procaspase-3 panels
  ○ Fig 5F Procaspase 3 panel

Author RB, with the agreement of FV and ES, responded to the concerns raised, stating that they did not agree with the concerns raised with Figs 1B, 2A, 2B, 2C, 2D, 5C, 5D, 5F and S2, and provided their own image analysis as well as some replicate experiment data. Regarding the concerns with Fig 5A, 5B and 5E, the author stated that unspecific bands were removed from the published panels. The author stated that the original data used to prepare the published figures are no longer available due to these images being taken more than 15 years ago, and provided updated panels for Fig 5A, 5B and 5E using image data representing replicates of the experiments. In the absence of the underlying data, the concerns raised with Figs 1, 2, 5 and S1 cannot be resolved, and PLOS does not consider replicate or repeat experiment data alone sufficient to resolve the concerns raised with the published figures.

In light of the above concerns that question the reliability and integrity of the Figs 1, 2, 5 and S1 results, the *PLOS ONE* Editors retract this article.

RB and FV did not agree with the retraction. ES and SLN either did not respond directly or could not be reached.
